# Snacking Behavior and Association with Metabolic Risk Factors in Adults from North and South India

**DOI:** 10.1016/j.tjnut.2022.12.032

**Published:** 2023-01-06

**Authors:** Anjali Ganpule, Manisha Dubey, Himanshi Pandey, Nikhil Srinivasapura Venkateshmurthy, Rosemary Green, Kerry Ann Brown, Avinav Prasad Maddury, Rajesh Khatkar, Prashant Jarhyan, Dorairaj Prabhakaran, Sailesh Mohan

**Affiliations:** 1 Centre for Chronic Disease Control, New Delhi, India; 2 Public Health Foundation of India, New Delhi, India; 3 London School of Hygiene and Tropical Medicine, London, UK; 4 University of Exeter, Exeter, UK; 5 Deakin University, Melbourne, Australia

**Keywords:** adults, India, metabolic risk, snacking behavior

## Abstract

**Background:**

Snacks are increasingly contributing to daily diets around the world. Studies from high-income countries have demonstrated the link between snack consumption and metabolic risk factors, but there are very few studies from low- and middle-income countries.

**Objectives:**

The objective of this study was to assess snack behavior and its associations with metabolic risk factors in Indian adults.

**Methods:**

Adults from the UDAY study (October 2018–February 2019, *n* = 8762) from rural and urban Sonipat (North) and Vizag (South) India were studied for snack consumption (food frequency questionnaire), demographic factors, including age, sex, etc. and metabolic risk factors, including BMI, waist circumference, fat percentage, plasma glucose, and blood pressure. We compared snack consumption by categories of sociodemographic factors (Mann–Whitney *U* test, Kruskal–Wallis test) and studied the likelihood for metabolic risk (logistic regression analysis).

**Results:**

Half of the study participants were women and resided in rural locations. Savory snacks were the most preferred; 50% of the participants consumed them 3–5 times/wk. Participants preferred to purchase out-of-home prepared snacks and eat them at home (86.6%) while watching television (69.4%) or with family/friends (49.3%). The reasons for snacking were hunger, craving, liking, and availability. Snack consumption was higher in Vizag (56.6%) than in Sonipat (43.4%), among women (55.5%) than men (44.5%), and the wealthiest; it was similar in rural–urban locations. Frequent consumers of snacks had 2 times higher likelihood for having obesity (OR: 2.22; 95% CI: 1.51, 3.27) central obesity (OR: 2.35; 95% CI: 1.60, 3.45), and higher fat percentage (OR: 1.92; 95% CI: 1.31, 2.82) and higher fasting glucose levels (r=0.12 (0.07–0.18) than consumers who consumed snacks rarely (all *P* ≤ 0.05).

**Conclusions:**

Snack (savory and sweet) consumption was high among adults from sexes in both urban and rural locations of north and south India. This was associated with higher risk of obesity. There is a need to improve the food environment by promoting policies for ensuring healthier food options to reduce snacking and associated metabolic risk.

## Introduction

A snack is a food, usually smaller than a meal, and consumed in-between meals [1]. There are both traditional and modern snack foods either prepared out of the home or at home. Studies in low- and middle-income countries show that with rapid urbanization, the economic and lifestyle transition, consumption of out-of-home prepared snacks has increased, and there is an increased demand for packaged and ready-to-eat foods [2–4]. Studies on Indian dietary patterns report an increase in consumption of foods high in fat and sugar [5–7]. There is increased demand for packaged snacks and beverages in India [8]. Comparison between different countries shows that Indian snacks can be unhealthy because of high sodium content, energy density, total sugar content, and use of poor-quality edible oil [9]. Consumption of such foods is adding to empty calories [10] and is associated with high BMI, increased waist circumference [11], elevated levels of plasma glucose, and blood pressure [12, 13]. The existing literature is limited to overall consumption and does not specifically bring out the characteristics of snack consumption in India. The country is facing public health problem of micronutrient deficiencies and diet-related metabolic disorders [14–16]. It needs to prioritize good quality nutrition rather than just adequate calories and reduce unhealthy snack consumption. Thus, we need focused studies to explore patterns of snack consumption behavior in India and the reasons behind this to come up with solutions for curbing them [8]. The snack consumption data will help to plan strategies to achieve diets as guided by Eat Right India [17] and the EAT-Lancet commission, which will bring together the health priorities of diets as well as those for sustainability of the environment [18]. The current study addresses above research gaps by exploring: *1*) snacks (fruits, savory snacks, sweet snacks, and beverages) consumed by adults; *2*) comparison of snack consumption by individual factors, such as sociodemographic, EI, and physical activity, and external factors, such as place of residence (rural and urban), and comparison of 2 geographically and culturally diverse states (Andhra Pradesh and Haryana); *3*) cross-sectional associations of snack consumption with metabolic risk factors, such as BMI, waist circumference, body fat, glycemia, and blood pressure.

## Methods

### Study design

The analysis presented in this paper is based on data collected from adult male and female participants aged ≥30 y and above from urban and rural households in Sonipat (north India) and Vizag (south India). The snack consumption and behavior data were collected under the Sustainable and Healthy Food Systems project between August 2020 and February 2021. The data on dietary intake, physical activity, and metabolic risk factors were from the UDAY study, a community-based diabetes and hypertension prevention and management program that was conducted from October 2018 to February 2019. The detailed methodology of the UDAY study has been published elsewhere [19].

### Ethics

Ethics approval was obtained from the Institutional Ethics Committee of the Public Health Foundation of India and Centre for Chronic Disease Control, New Delhi (IRB No: IRB00006330). Participants willing to participate and who provided informed written consent were included in the study.

### Measurements

All the measurements were carried out by trained research staff, entered in a computer-assisted personal interview platform, and were closely monitored for quality.

### Demographics

Information on residence (urban or rural), site and state (Sonipat, Haryana or Vizag, Andhra Pradesh), age, sex, and employment status were collected through a pretested questionnaire.

### Wealth index

The wealth index was constructed using principal component analysis based on household facilities and asset data, separately for rural and urban households [20]. It was based on the ownership of 12 household assets (radio, television, computer, telephone, refrigerator, bicycle, scooter, car, washing machine, sewing machine, house, and land), and 5 key housing characteristics (water supply, type of toilet and whether it is shared, cooking fuel, housing material, and source of lighting). The first component in the principal component analysis was extracted and divided into quintiles—the first quintile being the poorest and the fifth being the richest.

### Daily EI and physical activity

The daily calorie consumption of the participants was calculated using data collected in the UDAY study, based on the amount and frequency of consumption of 23 different food groups assessed through food frequency questionnaires. The daily calories consumed were calculated using reference values for raw food from the Indian Food Composition Table [21] and for cooked food from Agharkar Research Institute [22].

The physical activity was measured using the WHO Global Physical Activity Questionnaire [23] as the duration (in minutes) of mild (walking, standing, and sitting), moderate (brisk walking, dancing, and gardening), and intense (hiking, running, and cycling) activities. These were adjusted for sleep time and total activity duration was calculated.

### Snack food consumption

Participants were asked about the frequency, serving size, and quantity of 10 types of snacks consumed in the last month using a food frequency questionnaire. Participants were also asked to report snacks purchased from outside the home and the cost per serving in Indian rupees. The foods were as follows: *1*) bakery products (patties, khari, etc.); *2*) packaged salty snacks (chips, kurkure, etc.); *3*) fried snacks (vada, samosa, etc.); *4*) biscuits; *5*) instant noodles (pasta, noodles, etc.); *6*) sweet snacks (rasgulla, laddoo, etc.); *7*) sugar-sweetened and/or aerated beverages (cola, pepsi, etc.); 8) tea/coffee; *9*) sugar-sweetened fruit juice; and *10*) fruits. These were further grouped into 3 food groups, which were used for analysis namely, snacks/unhealthy food (savory and sweets 1–6); beverages [7–9]; and fruits/healthy food [10].

### Snacking behavior and preference

The snacking behavior of the participants was assessed for consumption, timing, place preferred for purchasing, and eating snacks. The questionnaire designed for this study was based on previous studies on Indian snacking behavior and preference patterns [24–26]. To understand the preferences of participants related to activities done while eating snacks, brand preferences, factors, and top reasons considered for choosing snacks, multiple choice questions were designed. The participants were asked to select 3 options of all and rank them in the order of preference.

### Metabolic risk factors

Body weight, height, and waist circumference were measured following standard procedures by trained field staff in participants’ homes. BMI was calculated and participants were classified as having BMI in the underweight (<18.5 kg/m^2^)/normal (18.5–24.9 kg/m^2^)/overweight or obese (≥25 kg/m^2^) category as per WHO guidelines [27]. Using WHO waist circumference cut-offs, participants were classified as having a normal (male ≤ 94 cm, female ≤ 80 cm) or central obesity (male >94 cm, female >80 cm) [28].

Body fat was measured using a Tanita body composition analyzer and participants were categorized using the following cut-offs; men >25% and women >35% [29]. Blood was obtained from fasting participants for measurement of HbA1c and levels of plasma glucose. Blood pressure was measured using standardized protocol by trained staff. All measurements were made using a Cobas 311 autoanalyzer and Roche Diagnostics reagents. Plasma glucose was estimated using Hexokinase method. HbA1c was assayed by high-performance liquid chromatography method using reagents from Bio-Rad Laboratories, Hercules. Those with fasting blood sugar of ≥126 mg/dL or HbA1c of ≥6.5% were categorized as having hyperglycemia [30]. Hypertension was measured using standardized method [31] and validated instrument: OMRON JPN1 (HEM-7200-AP3). Those with systolic blood pressure of ≥140 mmHg or diastolic blood pressure of ≥90 mmHg or no treatment were categorized as having hypertension [31].

### Statistical analysis

The data were tested for normality and skewed variables were presented as median, interquantile range. Data representing age, daily EI, physical activity, cost, and metabolic risk factors were presented as categorical data. All data related to snack behavior, including timing, factors, top reasons, etc. were summarized as frequency and percentages. We compared proportion of participants consuming snack and frequency of total snack consumption by categories of sociodemographic factors and metabolic risk using the Mann–Whitney *U* test for 2 categories and Kruskal–Walli’s test for >2 categories. We did multiple comparisons using Bonferroni corrections to avoid inflation in *P* values. Fruits (healthy) and total snacks (unhealthy) consumption were compared (using proportion test) by top reasons for snack consumption. The cost of different foods was compared and their association with consumption was studied. We also analyzed the association of total snack consumption and beverages with metabolic risk factors using multivariable linear regression models. We compared the likelihood for metabolic risk among participants consuming snacks rarely and frequently using logistic regression analysis. We adjusted the regression models for individual (age, sex, wealth index, employment, daily EI, and physical activity) and external (place of residence and state) factors. These factors were tested for collinearity, as the variance inflation factors were <10, there was an absence of multicollinearity. We have used 2-tailed tests with a 5% level of significance. The statistical analysis was conducted using Stata 16.1 (Stata Corp)/SPSS 22 (IBM, India).

## Results

### Background characteristics

The study presents data on 8762 participants who had information on snacking behavior (100%) and metabolic risk factors (90%) (data completeness BMI 97%, fat% 99%, waist circumference >99%, hyperglycemia 87%, and hypertension 100%). The mean age of the participants was 52.4 ± 11.7 y, 56.1% of participants were from rural locations and 56.5% were women and about half of the participants were from Sonipat (52.0%) (Table 1). Higher proportion of employed participants consumed snacks rather than those who were unemployed, retired, or managing homes. Around 45.6% (95% CI: 44.6%, 46.7%) had BMI in the overweight and obese category, 43.9% (95% CI: 42.9%, 45.1%) had waist circumference in central obesity category, 47.1% (95% CI: 46.1%, 48.2%) had body fat percent in the higher category, 13.9% (95% CI: 13.1%, 14.7%) of participants had hyperglycemia and 45.5% (95% CI: 44.8%, 47.0%) had hypertension.

### Fruit and snack consumption

**Figure 1:**
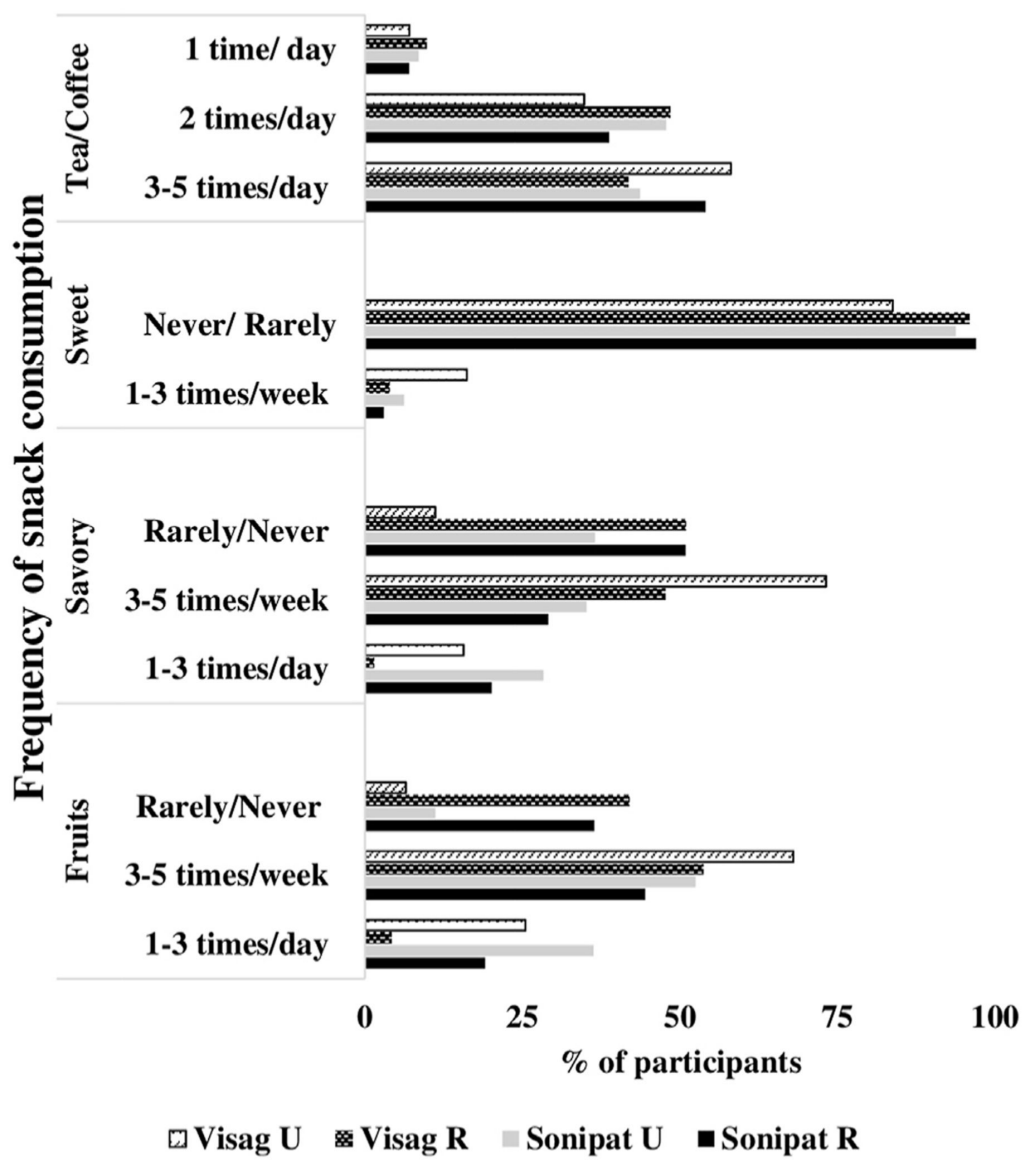
The figure shows the frequency of various snack food consumed by the place of residence (rural–urban) in Sonipat and Vizag. The various snack foods were: fruits, savory snacks, sweet snacks, and tea/coffee. Their frequency is listed as per the consumption. i.e., either daily, weekly, or rarely.

Fruits were the second most frequently consumed snack after savory snacks. Only 20%–30% participants consumed fruits daily, whereas almost half of the participants consumed them weekly. Frequency of fruit consumption was lower in rural than urban locations (Figure 1). Savory snacks were the most frequently consumed snacks at all places. They were consumed everyday by around one-fourth of the participants from Sonipat; every week 3–5 times by ~30% participants. In Vizag, <10% of participants consumed savory snacks daily, whereas 60% of participants consumed them weekly. Of the savory snacks, the frequency of packaged salty snack consumption was higher than that of fried (results are not shown in the figure). Overall, the frequency of savory snack consumption was higher among urban participants than rural. The frequency of consumption of sweet snacks was relatively low; only 10% consumed them weekly, whereas ~90% of participants never or rarely consumed them.

### Beverage consumption

Although snacks generally refer to solid foods, we also asked for consumption of beverages, considering their high sugar content. Almost everyone consumed tea/coffee daily, half of the participants consumed them 3–5 times/day; whereas ~40% consumed them twice a day. Of the beverages, sweet fruit juices and soft drinks were hardly consumed by the participants, only 25% consumed them rarely (<4 times/mo).

### Calorie contribution of snacks

When we studied the calorie contribution of the snack foods (savory and sweet), we found that they contributed a median of 15.2% (IQR: 10.4–22.2) 184 kcal (IQR: 144–244) to the total daily calories (results are not shown in tables and figures). These percentages were higher in Sonipat (19.7% [IQR: 13.1%–27.6%]) than in Vizag (12.4% [IQR: 9.2%–16.6%]), in urban (16.7% [IQR: 11.6%–22.8%]) than in rural areas (14.0% [IQR: 9.6%–21.2%]), and in participants in the wealthiest (18.9% [IQR: 12.7%–27.0%]) compared with the poorest (13.5% [IQR: 9.5%–19.5%]) wealth index category (all *P* < 0.001).

### Cost of food

We also asked for the cost of snack foods. The cost one serve of savory foods was (₹13.1 [IQR: 7.1–24.0]), whereas it was (₹11.7 [IQR: 8.3–16.7]) for fruits. The cost of fruits was double in Vizag (₹16.7 [IQR: 8.3–20.0]) than in Sonipat (₹8.3 [IQR: 8.3–16.7]), whereas it was the similar for sweet snacks obtained outside of homes (₹20.1 [IQR: 10.2–20.3]). The cost of savory snacks was higher in Vizag (₹14.3 [IQR: 4.1–24.2]) than in Sonipat (₹10.1 [IQR: 7.1–20.3]). The cost of beverages was similar in Vizag (₹7.5 [IQR: 4.2–13.3]) and Sonipat (₹8.3 [IQR: 5.2–13.3]) (all *P* < 0.05). When we studied the association of cost with consumption of snack foods, the higher cost of fruits was associated with their lower consumption among participants belonging to the lowest wealth index (*r* = −0.06, *P* < 0.05). Again, higher cost of beverages was associated with their lower consumption in the rural areas *r* = −0.05, *P* value of <0.05. The cost was not associated with consumption for the savory and sweet snack foods (results are not shown in tables and figures).

### Snacking behavior and preferences

Participants were asked to report multiple factors, preferences, and reasons for snacking in general. The preferred reported timing for snacking was evening for participants from Sonipat and was morning for Vizag (Table 2). Most of the participants preferred to purchase snacks prepared out-of-home, mainly from street vendors. On the other hand, participants from rural Vizag preferred to consume snacks prepared at home. The preferred place to eat snacks was home, mainly with family members and friends or while watching television. Taste and cost were the commonly considered factors while choosing a snack. Participants from urban Vizag preferred local brands, whereas other participants from Vizag and Sonipat were unaware of the available brands. Participants reported craving (*an intense desire to consume a specific food*) and hunger as the top reasons for snacking followed by availability and liking (*important driver for food consumption*). When we cross-tabulated the above reasons with the type of snack consumed, we found that most of the participants preferred to eat snacks (savory and sweet) rather than fruits (*P* < 0.01 for all) (Table 3). There were a few exceptions, e.g., a higher proportion of urban participants preferred to consume fruits (62.0%) over snacks (6.9%) when hungry. Also, a higher proportion of participants from Sonipat preferred to consume fruits (32.5%) over snacks (5.3%) because of liking or availability.

### Association of snack consumption and sociodemographic factors (exposure) with metabolic risk

#### Univariate analysis

**Figure 2:**
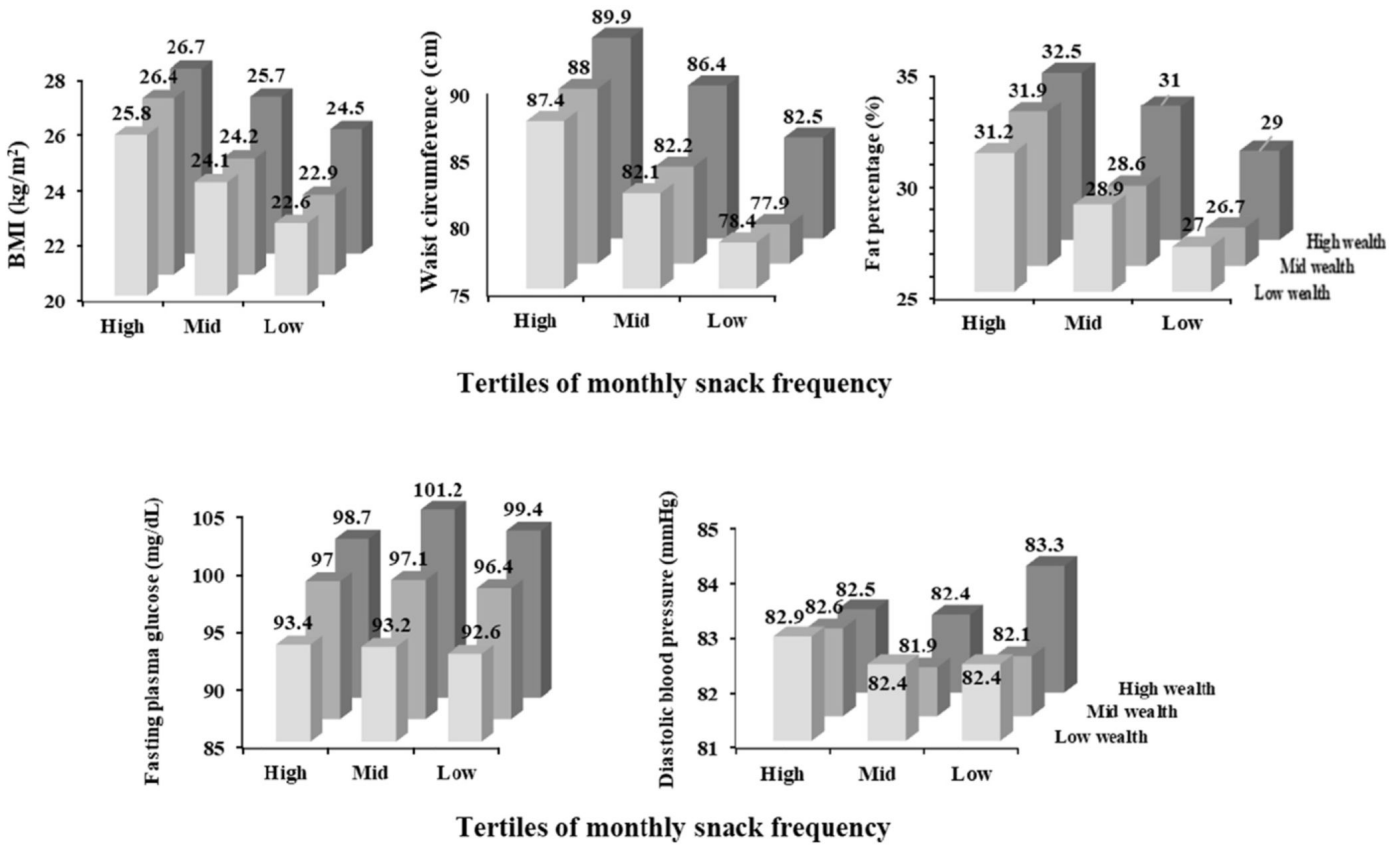
This figure shows association of snack consumption (savory + sweet) and wealth index with various metabolic risk factors. The snack consumption and wealth index were divided into tertiles of high, mid, and low. The metabolic factors were BMI, waist circumference, body fat percentage, plasma glucose, and diastolic blood pressure.

The proportion of participants and their frequency of consuming snacks was compared by various sociodemographic and metabolic risk factors (Table 1). Higher proportion of female participants, those belonging to older age groups and those with higher wealth index consumed snacks in higher frequency. Those residing in Vizag had a higher proportion of participants consuming snacks than those residing in Sonipat, and rural locations had a higher proportion of snack consumers than urban locations. Those with the highest snacking frequency and highest wealth index had the highest BMI, waist circumference and fat percent (Figure 2). Those with higher wealth index had higher fasting plasma glucose levels and diastolic blood pressure.

#### Multivariable analysis

We checked the associations of consumption of snacks (savory and sweet) and beverages independently with each of the metabolic risk factors adjusting for individual (age, sex, wealth index, employment, daily EI, and physical activity) and external (place of residence and state) factors (Table 4). We found that higher frequency of consumption of snacks was associated with most of the metabolic risk factors, including BMI, waist circumference, body fat percentage, glycemia, and systolic blood pressure, whereas diastolic blood pressure was not associated. Of these, BMI, waist circumference, and glycemia remained significantly associated after adjusting for individual and external factors.

Higher frequency of beverages consumption was associated with most of the metabolic risk factors (Supplemental Table 1). BMI and body fat percentage remained significantly associated even after adjusting for individual and external factors. Further, when we checked the associations using logistic regression, we found that those consuming snacks (savory and sweet) more 1–3 times daily had a twice higher likelihood of having a higher metabolic risk than those consuming snacks rarely or never (see Supplemental Tables 2 and 3).

## Discussion

This study brings out important evidence related to snack consumption behavior among rural and urban Indians. In this study, we assessed the consumption of out-of-home snacks and beverages in adults. Most of the participants consumed savory snacks, while sweet snacks (including fruits) were consumed less frequently. We found that sex, employment, wealth, place of residence, and cost are important determinants of snacking behavior. Higher snack consumption was associated with higher metabolic risk irrespective of individual as well as external factors. The risk was as high as twice in participants consuming snacks daily than those consuming snacks rarely. The study also reports important factors related to snack behavior, such as when and why people snack, which can contribute to increasing the limited information hitherto available in this area. The results of the study can potentially aid health promotion campaigns for advancing healthy and sustainable diets.

We found that the place of residence affects the snack consumption, related factors, and their association with metabolic risk. The place of residence characterizes the food environment components. Recently, snack and processed food consumption in rural India has increased [32]. This is because snacks and packaged foods are easily accessible and available in rural locations, whereas access to healthy foods, such as fruits, is limited [33]. These results highlight the importance of identifying the lacunas in specific components of food environment and targeting them to promote healthy food choices. Although it is known that components of snacks, such as refined carbohydrates, saturated and trans-fats, low fiber, high sugar, and salt content can add to the metabolic risk [34], community-based studies in India of these effects are limited. This study tries to address this gap and provide the relevant evidence.

The way snacks are consumed adds to the deteriorating effects because they are often consumed in increased portion sizes and, while doing sedentary activities, such as sitting at home [35] or watching television [36]. Our study found similar results as most of the participants preferred snack consumption at home while watching television. Additionally, there are many social and psychological cues to consuming snack foods [34]. These obesogenic environments need to be addressed to encourage healthy dietary behaviors (e.g., snacks, such as fruits or high-fiber foods, such as salads) and reduce metabolic risk [37].

We found that multiple factors guide snacking behavior. Our study shows that craving and hunger were the most reported factors for choice of unhealthy snacks, such as fried, packaged, and sweet snacks. Additionally, easy availability, liking, taste, and cost emerged as factors associated with greater unhealthy snacking. This supports previous qualitative and quantitative research, which also found hunger [24, 25], craving, liking, and availability [26] as common reasons for having snacks [38]. Studies have tried to identify different factors and reasons for having healthy and unhealthy foods. Healthy foods, such as fruits and vegetables, are less consumed as they are hard to store and costlier [39]. Although unhealthy snacks are consumed because of reasons, such as lifestyle, paucity of time, declining skills to prepare traditional Indian foods, and changing roles of women [40]. Studies also report the effect of urbanization [41] or income [42], or the parallel effect of both [43] on the choice of snacks. Our study found that the cost and economic status of the participants affected the consumption of healthy food, such as fruit, whereas cost did not affect consumption of unhealthy snack foods. This indicates the need to improve the affordability of healthy foods.

There have been some efforts on these lines in India. For instance, efforts, such as Rythu bazaars or farmer’s bazaars in Andhra Pradesh, where the vegetables and fruits come to market directly from farmers, eased the issues related to affordability and accessibility of fruits-vegetables and benefitted farmers [44]. Support for similar efforts at national levels could be considered to enhance the food environment.

The study had a few limitations. Participants were asked to report snacks purchased from outside the home and not all snacks consumed. However, there are chances that participants may have given mixed responses. The snack consumption and metabolic risk factors were measured 6 at different time points with a gap of ~6 mo. However, considering that dietary patterns among adults are relatively constant, it is unlikely to have impacted the findings.

The coronavirus disease 2019 (COVID-19) pandemic and the lockdown increased the consumption of processed comfort foods. For instance, a study in Mumbai, India, reported an increased preference for convenient and ready-to-eat foods with low preparation time during COVID-19 [45]. Although we did not have comparative before-after COVID-19 data on snack consumption, our study was conducted during early COVID-19 times (August to December 2020) that may have impacted our findings on snack consumption.

Worldwide there are varied guidelines available for snack consumption. For example, a review showed there are 136 snacking-specific recommendations from different countries, some provide advice on the quality of snacks and others focus on the frequency or nutrient composition [46]. In the Indian context, we need guidelines considering Indian snack consumption pattern. However, these details are lacking and our study helps fill this knowledge gap. In addition to these guidelines for consumers, there is a need to implement policy regulations to maintain the quality of snacks. Recently, efforts have been made in India to curb the consumption of snacks at the school level. There are restrictions on the sale of snacks on school and college campuses. Also, the Food Safety and Standards Authority of India, the national regulator has been initiating changes that have prompted higher standards in food and its quality in India. Food Safety and Standards Authority of India has limited the use of industrial trans-fatty acids (not >3% in all fats and oils) by January 2021 and not >2% by January 2022 [17]. We need a more comprehensive approach while formulating policies under different ministries. For example, subsidies to improve the quality of the food supply must also consider the health and nutrition aspects.

Globally, the need for a wider understanding of how the modern food system shapes dietary patterns and eating behavior is being highlighted. Our study partly addressed this gap in the Indian scenario. This helps to clear the interrelationship between food systems, food environments, diets and adverse public health and environmental factors, and the potential for better diets to lead to major improvements in public health and environmental sustainability [47, 48]. It is a 2-way process, encouraging people to make healthier food choices by educating them to consume fewer foods associated with metabolic disorders and making food environments healthier to foster healthier food choices.

In conclusion, the study reports high consumption of snacks in both urban and rural locations in north and south India. It brings out multiple factors associated with snacking that are important for planning targeted actions and public health interventions to encourage healthy diets. There is a need to improve the food environment by promoting policies for ensuring healthier food options to reduce snacking and associated metabolic risk.

## Supplementary Material

Supplementary data to this article can be found online at https://doi.org/10.1016/j.tjnut.2022.12.032.

Tables

## Figures and Tables

**Table 1:** Comparison of snack consumption by sociodemographic and metabolic risk factors

Sample characteristics	Categories	Participants *N* (%)	Snack consumers *n* (%)^2^	Snacking frequency times/mo Median (IQR)^3,4^
State	Sonipat	4558 (52.0)	2817 (43.4)	17 (8–30)
	Vizag	4204 (48.0)	3674 (56.6)^1^	8 (4–18)^1^
Residence	Rural	4915 (56.1)	3350 (51.6)	8 (4–16)
	Urban	3847 (43.9)	3141 (48.4)^1^	18 (10–30)
Age (y)	30–44	2514 (28.7)	1999 (30.8) ^Ref^	12 (5–24) ^Ref^
	45–59	3760 (42.9)	2782 (42.9)^1^	12 (5–26)^1^
	≥60	2488 (28.4)	1710 (26.3)^1^	12 (5–30)^1^
Sex	Male	3813 (43.5)	2886 (44.5)	12 (6–26.3)
	Female	4949 (56.5)	3605 (55.5)^1^	12 (4–24)^1^
Wealth index	Poorest	1697 (19.4)	1283 (19.8) ^Ref^	8 (4–18) ^Ref^
	Poor	1732 (19.8)	1396 (21.5)^1^	10 (4–18)^1^
	Middle	1832 (20.9)	1393 (21.5)^ns^	12 (4–22)^1^
	Rich	1745 (19.9)	1257 (19.4)^1^	16 (8–30)^1^
	Richest	1756 (20.0)	1162 (17.9)^1^	18 (8–30.3)^1^
Employment	Unemployed	93 (1.1)	63 (1.0) ^Ref^	13 (8–30) ^Ref^
	Housewife	3409 (38.9)	2363 (36.4)^ns^	15 (8–30)^ns^
	Retired	785 (9.0)	584 (9.0)^ns^	16 (8–30)^ns^
	Employed	4473 (51.1)	3480 (53.6)^ns^	10 (4–20)^ns^
Daily energy intake (EI)	Low (<2000 kcal/d)	8386 (95.7)	6175 (95.1)	12 (5–25)
	High (≥2000 kcal/d)	376 (4.3)	316 (4.9)^1^	10 (4–25.8)
Daily physical activity	Mild (<600 min/d)	3079 (35.2)	2003 (30.9) ^Ref^	16 (8–30) ^Ref^
	Moderate (600–792.8 min/d)	2769 (31.6)	2078 (32.0)^1^	13 (7–26)^1^
	Intense (≥792.8 min/d)	2912 (33.2)	2409 (37.1)^1^	8 (4–17)^ns^
BMI categories	Underweight (<18.5 kg/m^2^)	814 (9.6)	596 (9.5) ^Ref^	8 (4–16) ^Ref^
	Normal (18.5–24.9 kg/m^2^)	3816 (44.8)	2786 (44.3)^ns^	11 (4–21)^1^
	Overweight/obese (≥25 kg/m^2^)	3887 (45.6)	2913 (46.3)^ns^	15 (8–30)^1^
Waist circumference	Normal (M ≤94 cm, F ≤80cm)	4878 (56.1)	3655 (56.7)	10 (4–20)
	Central obesity(M >94 cm, F>80 cm)	3820 (43.9)	2792 (43.3)^1^	16 (8–30)^1^
Fat percentage	Low fat% (M ≤25, F ≤35)	4584 (52.8)	3335 (51.9)	9 (4–20)
	High fat% (M >25, F >35)	4085 (47.1)	3086 (48.1)^1^	15 (8–30)^1^
Plasma glucose	Normoglycemic (≤125 mg/dL)	6603 (86.1)	4897 (85.4)	12 (4–24)
	Hyperglycemic (>125 mg/dL)	1065 (13.9)	840 (14.6)^1^	16 (8–30)^1^
	(>125 mg/dL or HbA1c ≥6.5%)			
Blood pressure	Normotensive(<140/<90) mmHg)	4774 (54.5)	3577 (55.1)	11 (4–23)
	Hypertensive (≥140/≥90) mmHg)	3988 (45.5)	2914 (44.9)^1^	14 (7–30)^1^

1 *P ≤* 0.05, ns *P* > 0.05.

2 Comparisons column 4- *t*-test (2 categories) or ANOVA (>2 categories) to compare the proportion of participants consuming snacks by state, residence, age, and so on.

3 Monthly frequency of snacks (savory + sweet) consumption.

4 Column 5- Mann–Whitney *U* test (for 2 groups) by state, residence, age group (category 1 = reference), wealth index, employment (compared with unemployed), and so on.

**Table 2:** Factors, preference, and reasons for snack consumption

	Snack consumption^1^			Vizag (*n* = 4204) *n* (%)			Sonipat (*n* = 4558) *n* (%)		
	Preferences^2^ *n* (%)	Residence		Rural (*n* = 2501)	Urban (*n* = 1703)		Rural (*n* = 2414)	Urban (*n* = 2144)	
	**Time**	Morning		85 (4.5)	394 (24.2)		672 (60.8)	909 (85.2)	
		Afternoon		352 (18.7)	218 (13.4)		667 (60.4)	242 (22.7)	
		Evening		1797 (95.3)	1572 (96.7)		303 (27.4)	339 (31.8)	
		Late-night		286 (15.2)	216 (13.3)		45 (4.1)	9 (0.8)	
		Midnight		1 (0.1)	(0.0)		(0.0)	(0.0)	
	**Place preferred to eat**	Home		1666 (88.3)	1269 (78.0)		948 (85.8)	1006 (94.3)	
		Work place		52 (2.8)	302 (18.6)		119 (10.8)	49 (4.6)	
		Out of home		168 (8.9)	55 (3.4)		38 (3.4)	12 (1.1)	
	**Source**	Home		1697 (90.0)	495 (30.4)		91 (8.2)	156 (14.6)	
		Street vendor		1050 (55.7)	1110 (68.3)		909 (82.3)	900 (84.3)	
		Restaurant		13 (0.7)	75 (4.6)		169 (15.3)	158 (14.8)	
	**Activities performed**	Working		22 (1.2)	223 (13.7)		150 (13.6)	47 (4.4)	
		Watching TV		1349 (71.5)	1389 (85.4)		551 (49.9)	754 (70.7)	
		Travelling		122 (6.5)	785 (48.3)		123 (11.1)	66 (6.2)	
		With family/friends		1249 (66.2)	1421 (87.4)		254 (23.0)	219 (20.5)	
	**Factors considered**	Price		832 (44.1)	1311 (80.6)		243 (22.0)	544 (51.0)	
		Brand		259 (13.7)	990 (60.9)		368 (33.3)	255 (23.9)	
		Taste		1797 (95.3)	1397 (85.9)		974 (88.1)	707 (66.3)	
		Advertisement		49 (2.6)	310 (19.1)		40 (3.6)	(0.0)	
		Free-gift/offer		290 (15.4)	56 (3.4)		106 (9.6)	52 (4.9)	
		Sugar/salt/fat		274 (14.5)	450 (27.7)		75 (6.8)	58 (5.4)	
		Others		4 (0.2)	65 (4.0)		1 (0.1)	5 (0.5)	
	**Brand preferred**	Local		717 (38.0)	786 (48.3)		149 (13.5)	307 (28.8)	
		National		125 (6.6)	565 (34.7)		319 (28.9)	66 (6.2)	
		Foreign		2 (0.1)	7 (0.4)		3 (0.3)	(0.0)	
		No preference		472 (25.0)	132 (8.1)		238 (21.5)	190 (17.8)	
		Unaware		570 (30.2)	136 (8.4)		396 (35.8)	504 (47.2)	
	**Top reasons**	Hunger		996 (52.8)	1025 (63.0)		455 (41.2)	260 (24.4)	
		Craving		1314 (69.7)	1399 (86.0)		262 (23.7)	282 (26.4)	
		Availability		417 (22.1)	1006 (61.9)		718 (65.0)	478 (44.8)	
		Like it		995 (52.8)	664 (40.8)		432 (39.1)	515 (48.3)	
		It is healthy		83 (4.3)	357 (22.0)		145 (13.1)	12 (1.1)	
		Others		61 (3.2)	125 (7.7)		(0.0)	6 (0.6)	

1 Snacks (savory + sweet) consumption.

1 All the above questions are multiple choice question and therefore total percent does not add to 100.

**Table 3:** Comparison of fruit and snack consumption by top reasons for consuming snacks

Top reasons for snack consumption			Fruits			Snacks^2^		
			*n* (*N*)	Percent (95% CI)		*n* (*N*)	Percent (95% CI)^3^	
**Craving**	**State**	Sonipat	142/544	26.1 (22.4–29.8)		147/544	27.0 (23.3–30.8)^ns^	
		Vizag	173/2713	6.4 (5.5–7.3)		390/2713	14.4 (13.1–15.7)^1^	
	**Residence**	Rural	181/1576	11.5 (9.9–13.1)		402/1576	25.5 (23.4–27.7)^1^	
		Urban	134/1681	8.0 (6.7–9.3)		135/1681	8.0 (6.7–9.3)^ns^	
	**Sex**	Female	185/1741	10.6 (9.2–12.1)		293/1741	16.8 (15.1–18.6)^1^	
		Male	130/1516	8.6 (7.2–10.0)		244/1516	16.1 (14.2–17.9)^1^	
**Hunger**	**State**	Sonipat	125/715	17.5 (14.7–20.3)		174/715	24.3 (21.2–27.5)^1^	
		Vizag	42/2021	2.1 (1.5–2.7)		154/2021	7.6 (6.5–8.8)^1^	
	**Residence**	Rural	87/1451	6.0 (4.8–7.2)		239/1451	16.5 (14.6–18.4)^1^	
		Urban	80/1285	62.0 (49.0–75.0)		89/1285	6.9 (5.5–8.3)^ns^	
	**Sex**	Female	105/1560	6.70 (5.5–8.0)		205/1560	13.1 (11.5–14.8)^1^	
		Male	62/1176	5.3 (4.0–6.5)		123/1176	10.5 (8.7–12.2)^1^	
**Liking**	**State**	Sonipat	308/947	32.5 (29.5–35.5)		92/947	9.70 (7.8–11.6)^1^	
		Vizag	52/1659	3.1 (2.3–4.0)		348/1659	21.0 (19.0–22.9)^1^	
	**Residence**	Rural	128/1427	9.0 (7.5–10.5)		195/1427	13.7 (11.9–15.4)^1^	
		Urban	232/1179	19.7 (17.4–21.9)		245/1179	20.8 (18.5–23.1)^ns^	
	**Sex**	Female	178/1420	12.5 (10.8–14.3)		221/1420	15.6 (13.7–17.4)^1^	
		Male	182/1186	15.3 (13.3–17.4)		219/1186	18.5 (16.3–20.7)^1^	
**Availability**	**State**	Sonipat	389/1196	32.5 (29.9–35.2)		10/1196	0.8 (0.0–1.4)^1^	
		Vizag	8/1423	0.6 (0.2–10.0)		489/1423	34.4 (31.9–36.8)^1^	
	**Residence**	Rural	206/1135	18.1 (15.9–20.4)		287/1135	25.3 (22.8–27.8)^1^	
		Urban	191/1484	12.9 (11.2–14.6)		212/1484	14.3 (12.5–16.1)^ns^	
	**Sex**	Female	198/1402	14.1 (12.3–15.9)		261/1402	18.6 (16.6–20.7)^1^	
		Male	199/1217	16.4 (14.3–18.4)		238/1217	19.6 (17.3–21.8)^1^	

1 *P* ≤ 0.05, ns *P* > 0.05.

2 Snacks (savory + sweet) consumption.

3 To compare proportions of participants who consumed fruits or snacks according to their top reason for snacking by state, residence, and sex the *z*-test was used.

**Table 4:** Multivariable analysis of snack^2^ consumption with metabolic risk

Metabolic risk factors	Unadjusted β (95% CI)	Adjusted for individual factors^3^ β (95% CI)	Adjusted for external factors^4^ β (95% CI)
BMI, kg/m^2^	0.04 (0.04–0.05)^1^	0.02 (0.01–0.03)^1^	0.01 (0.00–0.01)^1^
Waist circumference, cm	0.13 (0.11–0.15)^1^	0.05 (0.04–0.07)^1^	0.02 (0.00–0.04)^1^
Body fat percentage, %	0.06 (0.04–0.07)^1^	0.03 (0.02–0.04)^1^	0.01 (0.00–0.02)^ns^
Plasma glucose, mg/dL	0.17 (0.12–0.22)^1^	0.12 (0.07–0.18)^1^	0.07 (0.02–0.12)^1^
Systolic blood pressure, mmHg	0.06 (0.03–0.09)^1^	0.02 (0.00–0.05)^ns^	0.01 (–0.02–0.03)^ns^
Diastolic blood pressure, mmHg	0.01 (−0.01–0.02)^ns^	0.00 (−0.01–0.02)^ns^	−0.01 (−0.02–0.01)^ns^

1 *P* ≤ 0.05, ns *P* > 0.05.

2 Snacks (savory + sweet) consumption.

3 Individual factors: age, sex, wealth index, employment, daily EI, and physical activity.

4 External factors: state and place of residence (rural–urban).

## Data Availability

Data described in the manuscript, codebook, and analytical code will be made available upon reasonable request to the corresponding author.
