# Supplementary material for: Snacking Behavior and Association with Metabolic Risk Factors in Adults from North and South India
Source: J Nutr. Author manuscript; Available in PMC 2024 Jul 30. (PMC7616315; doi:10.1016/j.tjnut.2022.12.032)
Supplement: Tables [file EMS197575-supplement-Tables.zip › 1-s2.0-S0022316623005059-mmc1.docx]

**Supplementary Table 1: Multivariable analysis of beverages consumption with metabolic risk**

| **Metabolic risk factors** | **Unadjusted**  **β (95% CI)** | **Adjusted for individual factors^2^**  **β (95% CI)** | **Adjusted for external factors^3^**  **β (95% CI)** |
| --- | --- | --- | --- |
| **BMI, kg/m^2^** | 0.01(0.00-0.01)^1^ | 0.00(-0.01-0.00)^1^ | 0.00(-0.01-0.00)^1^ |
| **Waist circumference, cm** | 0.02(0.01-0.03)^1^ | 0.00(-0.01-0.00)^ns^ | -0.01(-0.01-0.00)^ns^ |
| **Body fat percentage, %** | 0.01(0.00-0.02)^1^ | -0.01(-0.02--0.01)^1^ | -0.01(-0.01-0.00) ^1^ |
| **Plasma glucose, mg/dL** | -0.04(-0.06--0.01)^1^ | -0.03(-0.06--0.01)^1^ | -0.01(-0.03-0.01)^ns^ |
| **Systolic blood pressure, mmHg** | 0.03(0.01-0.04)^1^ | 0.00(-0.01-0.01)^1^ | -0.01(-0.02-0.00)^ns^ |
| **Diastolic blood pressure, mmHg** | 0.00(-0.01-0.00)^ns^ | 0.00(-0.01-0.00)^1^ | -0.01(-0.01-0.00)^ns^ |

^1^ P ≤ 0.05, ns P >0.05

^2^ Individual factors: age, sex, wealth index, employment, daily energy intake and physical activity

^3^ External factors: state and place of residence (rural-urban)
