# Supplementary material for: Snacking Behavior and Association with Metabolic Risk Factors in Adults from North and South India
Source: J Nutr. Author manuscript; Available in PMC 2024 Jul 30. (PMC7616315; doi:10.1016/j.tjnut.2022.12.032)
Supplement: Tables [file EMS197575-supplement-Tables.zip › 1-s2.0-S0022316623005059-mmc3.docx]

**Supplementary Table 3: Association of beverages consumption with metabolic risk**

| **Beverage consumption** | **High metabolic risk factors**  **n(%)** | **Low metabolic risk factors**  **n(%)** | **Unadjusted**  OR (95% CI) | **Adjusted for individual factors^2^**  OR (95% CI) | **Adjusted for external factors^3^**  OR (95% CI) |
| --- | --- | --- | --- | --- | --- |
| **BMI** | (≥25 kg/m^2^) n= 3887 | (<25 kg/m^2^) n= 4630 |  |  |  |
| 3-5 times/week | 324(8.3) | 369(8.0) | Reference | Reference | Reference |
| 1-2 times/day | 1631(42.0) | 2031(43.9) | 0.91(0.78, 1.08) ^ns^ | 0.89(0.74, 1.06) ^ns^ | 0.86(0.72, 1.03) ^ns^ |
| 3-5 times/day | 1932(49.7) | 2230(48.2) | 0.99(0.84, 1.16) ^ns^ | 0.90(0.76, 1.07) ^ns^ | 0.86(0.72, 1.03) ^ns^ |
| **Waist circumference** | (M >94cm, F >80cm)  n= 3820 | (M ≤94cm, F ≤80cm)  n= 4878 |  |  |  |
| 3-5 times/week | 259(6.8) | 447(9.2) | Reference | Reference | Reference |
| 1-2 times/day | 1670(43.7) | 2071(42.5) | 1.39(1.18, 1.64)^1^ | 1.17(0.97, 1.41) ^ns^ | 1.13(0.93, 1.36) ^ns^ |
| 3-5 times/day | 1891(49.5) | 2360(48.4) | 1.38(1.17, 1.63)^1^ | 1.12(0.93, 1.35) ^ns^ | 1.07(0.89, 1.30) ^ns^ |
| **Fat**  **percentage** | (M >25%, F >35%)  n= 4085 | (M ≤25%, F ≤35%)  n= 4584 |  |  |  |
| 3-5 times/week | 312(7.6) | 390(8.5) | Reference | Reference | Reference |
| 1-2 times/day | 1789(43.8) | 1938(42.3) | 1.15(0.98, 1.36) ^ns^ | 1.08(0.91, 1.29) ^ns^ | 1.06(0.88, 1.26) ^ns^ |
| 3-5 times/day | 1984(48.6) | 2256(49.2) | 1.10(0.94, 1.29) ^ns^ | 0.97(0.81, 1.15) ^ns^ | 0.93(0.78, 1.11) ^ns^ |
| **Glycemia** | (>125 mg/dL) n= 1065 | (≤125 mg/dL) n= 6603 |  |  |  |
| 3-5 times/week | 68(6.4) | 578(8.8) | Reference | Reference | Reference |
| 1-2 times/day | 490(46.0) | 2804(42.5) | 1.49(1.14, 1.94)^1^ | 1.45(1.10, 1.92)^1^ | 1.47(1.10, 1.95)^1^ |
| 3-5 times/day | 507(47.6) | 3221(48.8) | 1.34(1.02, 1.75)^1^ | 1.27(0.96, 1.68) ^ns^ | 1.26(0.95, 1.68) ^ns^ |
| **Blood pressure** | (≥140/≥90mmHg) n= 3988 | (<140/<90mmHg) n=4774 |  |  |  |
| 3-5 times/week | 307(7.7) | 410(8.6) | Reference | Reference | Reference |
| 1-2 times/day | 1780(44.6) | 1986(41.6) | 1.20(1.02, 1.41)^1^ | 1.13(0.95, 1.34) ^ns^ | 1.11(0.93, 1.32) ^ns^ |
| 3-5 times/day | 1901(47.7) | 2378(49.8) | 1.07(0.91, 1.25) ^ns^ | 1.01(0.85, 1.19) ^ns^ | 0.99(0.83, 1.17) ^ns^ |

^1^ P ≤ 0.05, ns P >0.05

^2^ Individual factors: age, sex, wealth index, employment, daily calorie intake and physical activity

^3^ External factors: state and place of residence (rural-urban)
