# Supplementary material for: Snacking Behavior and Association with Metabolic Risk Factors in Adults from North and South India
Source: J Nutr. Author manuscript; Available in PMC 2024 Jul 30. (PMC7616315; doi:10.1016/j.tjnut.2022.12.032)
Supplement: Tables [file EMS197575-supplement-Tables.zip › 1-s2.0-S0022316623005059-mmc2.docx]

**Supplementary Table 2: Association of snack consumption with metabolic risk**

| **Snack consumption^2^** | **High metabolic risk factors**  **n(%)** | **Low metabolic risk factors**  **n(%)** | **Unadjusted**  OR (95% CI) | **Adjusted for individual factors^3^**  OR (95% CI) | **Adjusted for external factors^4^**  OR (95% CI) |
| --- | --- | --- | --- | --- | --- |
| **BMI** | (≥25 kg/m^2^) n= 416 | (<25 kg/m^2^) n=953 |  |  |  |
| Rarely | 191(31.1) | 529(55.5) | Reference | Reference | Reference |
| 2-4 times/week | 168(57.4) | 353(37.0) | 1.32(1.03, 1.69)^1^ | 1.13(0.85, 1.49) ^ns^ | 1.07(0.80, 1.43) ^ns^ |
| 1-3 times/day | 57(11.5) | 71(7.5) | 2.22(1.51, 3.27)^1^ | 1.38(0.87, 2.18) ^ns^ | 1.39(0.85, 2.28) ^ns^ |
| **Waist circumference** | (M >94cm, F >80cm) n= 405 | (M ≤94cm, F≤80cm) n= 980 |  |  |  |
| Rarely | 182(44.9) | 547(55.8) | Reference | Reference | Reference |
| 2-4 times/week | 166(41.0) | 360(36.7) | 1.39(1.08, 1.78)^1^ | 1.18(0.88, 1.59) ^ns^ | 1.15(0.85, 1.56) ^ns^ |
| 1-3 times/day | 57(14.1) | 73(7.4) | 2.35(1.60, 3.45)^1^ | 1.62(1.00, 2.63) ^ns^ | 1.64(0.98, 2.76) ^ns^ |
| **Fat percentage** | (M >25%, F >35%) n= 429 | (M ≤25%, F≤35%) n= 953 |  |  |  |
| Rarely | 203(47.3) | 523(54.9) | Reference | Reference | Reference |
| 2-4 times/week | 170(39.6) | 355(37.3) | 1.23(0.97, 1.58) ^ns^ | 1.11(0.84, 1.45) ^ns^ | 1.06(0.80, 1.42) ^ns^ |
| 1-3 times/day | 56(13.1) | 75(7.9) | 1.92(1.31, 2.82)^1^ | 1.19(0.76, 1.86) ^ns^ | 1.32(0.81, 2.16) ^ns^ |
| **Glycemia** | (>125 mg/dL) n= 111 | (≤125 mg/dL) n= 1094 |  |  |  |
| Rarely | 49(44.1) | 594(54.3) | Reference | Reference | Reference |
| 2-4 times/week | 49(44.1) | 408(37.3) | 1.46(0.96, 2.21) ^ns^ | 1.30(0.84, 2.02) ^ns^ | 1.43(0.90, 2.25) ^ns^ |
| 1-3 times/day | 13(11.7) | 92(8.4) | 1.71(0.89, 3.28) ^ns^ | 1.15(0.56, 2.36) ^ns^ | 1.63(0.75, 3.54) ^ns^ |
| **Blood pressure** | (≥140/≥90mmHg) n= 531 | (<140/<90mmHg) n= 862 |  |  |  |
| Rarely | 269(50.7) | 463(53.7) | Reference | Reference | Reference |
| 2-4 times/week | 203(38.2) | 327(37.9) | 1.07(0.85, 1.34) ^ns^ | 0.99(0.77, 1.28) ^ns^ | 0.90(0.69, 1.18) ^ns^ |
| 1-3 times/day | 59(11.1) | 72(8.4) | 1.41(0.97, 2.05) ^ns^ | 1.04(0.67, 1.60) ^ns^ | 0.93(0.59, 1.49) ^ns^ |

^1^ P ≤ 0.05, ns P >0.05

^2^ Snacks (savory + sweet) consumption

^3^ Individual factors: age, sex, wealth index, employment, daily calorie intake and physical activity

^4^ External factors: state and place of residence (rural-urban)
